# The use of zeolite-based geopolymers as adsorbent for copper removal from aqueous media

**DOI:** 10.1098/rsos.211644

**Published:** 2022-03-09

**Authors:** Haci Baykara, Maria de Lourdes Mendoza Solorzano, Jose Javier Delgado Echeverria, Mauricio H. Cornejo, Clotario V. Tapia-Bastidas

**Affiliations:** ^1^ Facultad de Ingeniería Mecánica y Ciencias de la Producción, Escuela Superior Politécnica de Litoral, ESPOL, Campus Gustavo Galindo km 30.5 Vía Perimetral, Guayaquil, Ecuador; ^2^ Center of Nanotechnology Research and Development (CIDNA), Escuela Superior Politécnica de Litoral, ESPOL, Campus Gustavo Galindo km 30.5 Vía Perimetral, Guayaquil, Ecuador; ^3^ Departamento Ciencias Químicas y Ambientales, Facultad de Ciencias Naturales y Matemáticas, Escuela Superior Politécnica de Litoral, ESPOL, Campus Gustavo Galindo km 30.5 Vía Perimetral, Guayaquil, Ecuador

**Keywords:** geopolymer, adsorption, zeolites, wastewater, kinetics, adsorption isotherms

## Abstract

Copper has been proven to have hazardous effects on human beings depending on its concentration levels. Recently, there has been a growing interest in developing geopolymers using local industrial minerals and by-products. However, research on the adsorption of heavy metals by geopolymer based on mordenite-rich tuffs is still limited. The geopolymer adsorbents have been synthesized using natural Ecuadorian zeolite-rich tuffs containing quartz, mordenite calcite and amorphous content with 20.8%, 28.5%, 4.2% and 46.4%, respectively. The geopolymers showed a maximum compressive strength of 26.86 MPa for 28 d of curing time. In the present study, an Ecuadorian zeolite-based geopolymer's removal capacity on copper ions in aqueous solutions, varying concentration and contact time were tested. Kinetic models were developed using pseudo first-order, pseudo second-order and the Elovich model. The adsorption data, using Cu^2+^ concentrations from 20 to 160 ppm, at 25°C were described by the Langmuir and Freundlich isotherms. Linear coefficient of determination (*R*^2^) results show that the Langmuir model fits the best. The attained adsorption capacity of 52.63 mg g^−1^ demonstrates the low-cost geopolymer's effectiveness for this study and its competitiveness compared with other studies. Adsorption kinetics follows the pseudo second-order kinetics model at the lower initial concentration of Cu^2+^.

## 1. Introduction

Copper, a heavy metal, has long- and short-term adverse effects on human health, especially on the gastrointestinal system and the environment in general [1,2]. Copper is widely used in the photographic and electronic industries, power plants in general, and consequently is commonly found in wastewater. Therefore, its availability beyond a critical threshold in the environment is undoubtedly dangerous for human beings and animals. So, it is crucial to control and remove a significant amount of copper from water [3].

Adsorption processes are a feasible alternative due to their flexibility in design and operation, and, in many cases, they generate high-quality treated effluents. There are so many different adsorbents such as magnetic cellulose nanocomposites [4], magnetic chitosan nanocomposite [5] and Titan yellow-thiourea-formaldehyde [6] systems for the removal of different pollutants from different media. It is known that adsorption possesses a lot of potential for metals removal in wastewater treatment as it is efficient and not costly. Adsorption versatility permits a wide range of metal-incorporating adsorbents for metals removal. Iron-incorporating adsorbents are of interest for copper removal [7]. Several factors such as the size of the hydrated ions, free energy of hydration and metal ions activity may be responsible for this selectivity of adsorption [8]. On the other hand, a geopolymer is an amorphous material generated by the reaction of an aluminium silicate with an alkali hydroxide, usually NaOH [9,10]. Recently, there has been a growing interest in developing geopolymers using local industrial minerals and by-products for immobilization of dangerous elements as a possible solution to the struggle against heavy metal contamination [11–14].

The removal of copper on metakaolin, fly ash and zeolite-based geopolymers and other inorganic solids have been demonstrated in several studies [8,15–18]. Fixed bed trials were carried out to assess modified silica capability to selectively remove Cu^2+^ from a multi-component solution [19,20], by using boiler mud and ash to remove copper by adsorption and precipitation processes from metal refining water.

There has been little investigation done on the adsorption of heavy metals by mordenite-rich tuffs-based geopolymers to the best of our knowledge. In this case, the use of natural raw materials in geopolymer synthesis has been challenging, mainly due to their heterogeneity. As known, geopolymers are thermally [21], mechanically [22] and chemically [23] stable materials. Due to these facts, geopolymers are very important materials that could be used *in situ* and under extreme conditions for different applications, especially in developing countries [24–26].

To the best of our knowledge, this is the first study that presents natural zeolite-based geopolymers as adsorbents to remove copper from aqueous media. Considering the abundance of natural zeolites in the coastal region of Ecuador, using this raw material for a geopolymer-based adsorbent for copper removal is feasible and cost-effective. Zeolite-rich tuffs and corresponding geopolymers have been characterized by Fourier transform infrared spectroscopy (FTIR), X-ray diffraction (XRD) and scanning electron microscopy and energy dispersive spectroscopy (SEM-EDS) techniques. Besides, the mechanical properties of geopolymers concerning the curing time have also been determined. Hence, the main goal of this study is to evaluate the capacity of the Ecuadorian zeolite-based geopolymer for the removal of copper ions from aqueous solutions by varying concentrations and contact times.

## 2. Materials and methods

### 2.1. Preparation of the geopolymer

The Ecuadorian zeolite was pulverized in a ball mill to collect the fraction less than 60 µm. Subsequently, it was added to an activating solution composed of Na_2_SiO_3_ (Merck, density 1.35 g ml^−1^ at 20°C, Na_2_O 7.5–8.5% and SiO_2_ 25.5–28.5%) and 10 M NaOH (Merck-Millipore, 99% purity) at a ratio of 2.5 : 1. The solution was mixed with zeolite at a ratio of 0.45 ml g^−1^ and stirred for 2 min. The resulting mixture was poured in 5 × 5 × 5 cm wooden moulds, covered with plastic bags and placed in an oven at 60°C for 24 h. Finally, the geopolymer cubes were left at room temperature (26 ± 2°C) for an additional 9 d (G-10), 16 d (G-17) and 27 d (G-28) before the compressive strength tests were carried out. Geopolymers have been prepared according to the previous papers published in the literature [9,10].

### 2.2. Characterization of the geopolymer

For structural stability, compressive strength tests were performed to assess the zeolite-based geopolymer's strength, using the ASTM C109/C109M-16a standard method [27] and a Shimadzu UTM-600KN universal testing machine. Prior to testing, geopolymer blocks labelled as G-10, G-17 and G-28, were sanded, sized (46.86 × 50.52 × 46.24 mm, 48.30 × 50.60 × 46.80 mm and 51.78 × 47.45 × 45.62 mm, respectively) and analysed for compressive strength tests after a total of 10, 17 and 28 curing days. The tensile rupture strength values were 40.3875, 52.1888 and 66.0000 kN for G-10, G-17 and G-28, respectively.

For quantitative X-ray diffraction analysis, a PANalytical X'Pert PRO equipment was used at 30 mA, 45 kV and angular measurement range of 0–80 (2°Theta). According to the study reported by Baykara *et al.* [10], the peaks of the crystalline structures present in the samples were determined with X'Pert High Score Plus software.

The elemental composition of the zeolite-based geopolymer samples was analysed using dispersive energy spectrophotometry using an FEI-Inspect S scanning electron microscope. For this purpose, the samples were crushed, and a small portion was taken on a plate with a graphite sheet. The gold coating was applied for the high-resolution micrographs of geopolymer samples.

For the FTIR test, 2 mg of the zeolite-based geopolymer samples (previously dried at 60°C for 6 h) were mixed and homogenized with 200 mg KBr, in an agate mortar. A pressure of 9 MPa was applied for 7 min for the pellet preparation. A Spectrum 100 Perkin Elmer spectrophotometer was used for the testing, with an atmosphere of ultra-high purity (UHP) grade nitrogen, with a spectrum range between 4000 and 400 cm^−1^ and a resolution of 1 cm^−1^.

For the thermogravimetric and the differential scanning calorimetry analysis, 10.0 ± 0.5 mg of the geopolymer sample was weighed in a previously red hot burned capsule. The equipment used was a thermogravimetric calorimeter TA SDT Q600, with a nitrogen flow of 100 ml min^−1^ and a ramp of 10.00°C min^−1^ in a range between 25°C and 1000°C. The data were interpreted using Advantage TA Universal Analysis 4.5A software.

### 2.3. Adsorption experiments

For the adsorption experiments, the geopolymer samples were ground to a particle size of less than an average of 36 µm (volume weighted mean D [3,4]). The details about the particle size distribution analysis results can be seen in electronic supplementary material, figure S1. The tests were carried out in batch mode by duplicated. For that, the synthesized geopolymer was dried for 2 h at 100°C and weighed before each adsorption experiment. On the other hand, standard solutions of 100 and 250 ppm of Cu^2+^ were prepared for the batch mode experiments, and for the determination of the adsorption isotherm, the Cu^2+^ concentration varied between 20 and 160 ppm at a temperature of 25°C. First, 50 ml of copper solution was poured into glass flasks previously immersed in a water bath to stabilize the temperature. Subsequently, 0.1 g of geopolymer was added to each flask, gently homogenized and allowed to stand still for 2 h. The solution was immediately vacuum filtered using 0.45 µm filter paper and analysed by flame atomic absorption spectroscopy (FAAS). The quantity of the copper adsorbed onto the geopolymer samples has been determined quantitatively using the atomic absorption spectrometer (Thermo Scientific ICE 3000 Series).

For the kinetic analysis, flasks containing 100 ppm of Cu^2+^ in aqueous solution and adsorbent dose of 2 g l^−1^ were placed in a water bath at 25°C. The flasks were vacuum filtered using 0.45 µm filter paper at a specified time and analysed by FAAS.

Adsorption capacity *q* (mg g^−1^) and efficiency were determined with equations (2.1) and (2.2), respectively,

2.1 $$q=\frac{(C_{0}-C_{e})\;\times \;V}{m}$$

and

2.2 $$E=\frac{\left(C_{0}-C_{e}\right)}{C_{0}}\;\times 100\text{\%}\;$$

where *C*_0_ (mg l^−1^) is the initial concentration, *C_e_* (mg l^−1^) is the equilibrium concentration, *V* (l) is the volume of the solution and *m* is the mass of the adsorbent.

## 3. Results and discussion

### 3.1. Characterization of the geopolymer

#### 3.1.1. Quantitative X-ray diffraction

**Figure 1.**
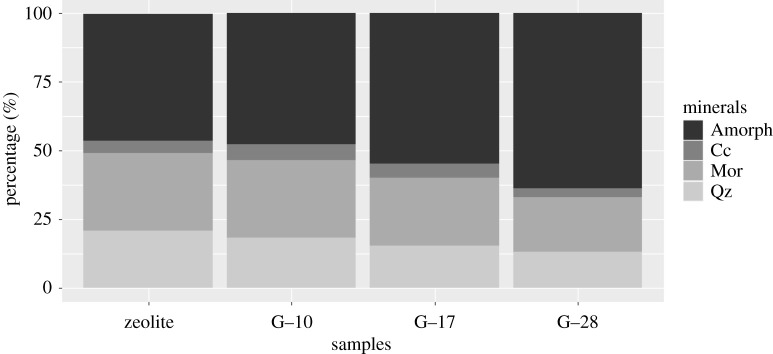
Quantitative X-ray diffraction (QXRD) analysis for crystalline and amorphous content of zeolite and geopolymer samples.

Figure 1 and electronic supplementary material, figure S2 present the XRD analyses of the zeolite and the three geopolymer samples prepared (G-10, G-17 and G-28) and cured for an additional 9, 16 and 27 d at room temperature. Quantitative mineral composition analysis has been done according to the Rietveld method [28]. The tuffs are mainly composed of mordenite, quartz and calcite. For the geopolymer samples, the amount of amorphous, ill-crystallized or non-crystallized phases increases with the curing time but decreases silica and mordenite. It seems that during the curing time, mordenite and quartz react with the alkaline activator to form an amorphous phase, mainly sodium aluminosilicate hydrate, as a result of geopolymerization reaction [29–31].

#### 3.1.2. Fourier transform infrared spectroscopy analyses

Both zeolite and geopolymer samples have been analysed in the wavenumber range from 4000 to 400 cm^−1^ (see electronic supplementary material, figure S3). Different bands were identified: (i) the band located around 3460 cm^−1^ is attributed to an asymmetric extension of H-O. Likewise, (ii) the band around 1636 cm^−1^ is due to H-OH bending and the adsorption of hydroxyl groups by excess alkali and water, respectively. Similarly, (iii) the vibration around 1040 cm^−1^ is credited to asymmetric stretching between Si-O-Si or Al-O-Si. Finally, (iv) the peaks around 1430 and 876 cm^−1^ are attributed to carbonate formed by exposure to atmospheric air [32,33]. The other peaks below 798 cm^−1^ are endorsed to different stretching modes, ring vibration of the structural network and bending between Si-O and Al-O bonds.

#### 3.1.3. Thermogravimetric analysis and differential scanning calorimetry

Table 1 and electronic supplementary material, figure S4 indicate the zeolite's thermogravimetric analysis and the geopolymers studied in this study. Between 0°C and 110°C, there is a significant loss of mass due to the material's porosity, which is evidenced in a more significant loss of surface water. In the range between 550°C and 750°C, the zeolite losses more mass compared with the geopolymer. This can be explained by the fact that the zeolite contains CaCO_3_, whose decomposition point is around 700–750°C [34], while the geopolymer, containing NaOH in excess, replaces Ca for Na, forming Na_2_CO_3_, which decompose in CO_2_ and Na_2_O at temperatures above 800°C [35]. Considering *ca* 14–15% total mass loss, it is evident that geopolymer samples are highly thermally stable. Due to this reality, this kind of material can be used as at high temperatures for different applications [36].

**Table 1.** Thermogravimetric analysis and differential scanning calorimetry (TGA-DSC) analysis (up to 1000°C) for zeolite and geopolymer samples.

sample	mass loss (%)						total mass loss (%)
temperature range (°C)	0–110	110–200	200–320	320–550	550–750	750–990	
zeolite	3.77	1.72	1.08	1.52	7.48	0.41	15.98
temperature range (°C)	0–150	150–212	212–350	350–600	600–700	700–750	
G-10	8.39	1.96	2.01	1.21	0.7	0.1	14.37
G-17	8.09	1.96	1.97	1.66	0.82	0.07	14.57
G-28	7.26	2.12	2.15	1.73	0.81	0.05	14.12

#### 3.1.4. Scanning electron microscopy and energy dispersive spectroscopy

**Figure 2.**
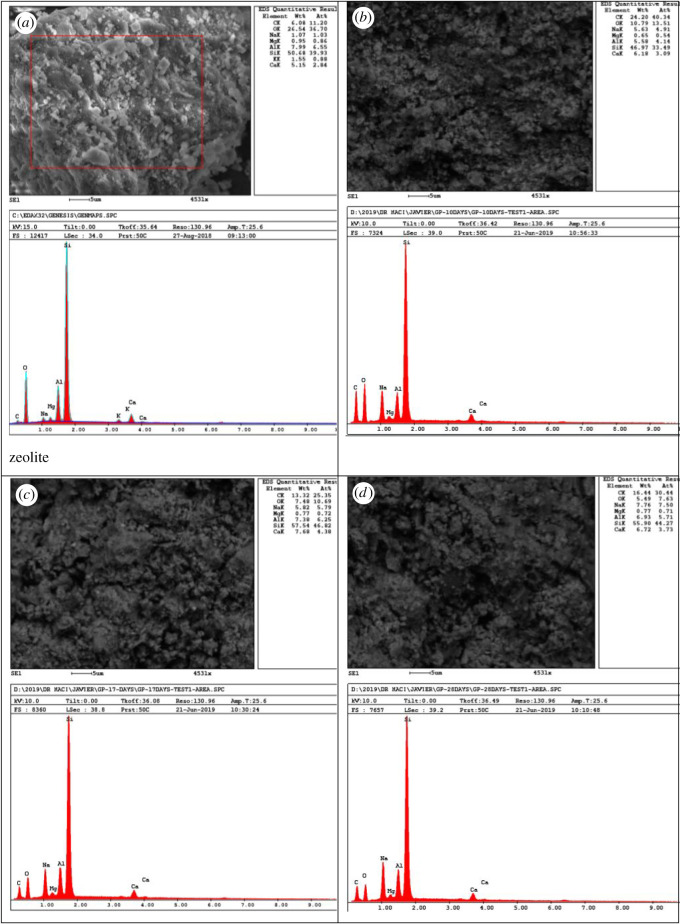
SEM-EDS images for (*a*) zeolite, (*b*) geopolymer cured for 9 d, (*c*) geopolymer cured for 16 d and (*d*) geopolymer cured for 27 d.

As seen in figure 2, there is an increase in sodium percentage in the geopolymer sample analysed. This change is attributed to NaOH and sodium silicate used for geopolymer synthesis. On the other hand, the increase in carbon percentage is due to the uptake of CO_2_ (carbonation) with unreacted and excess alkaline activators. Semi-quantitative elemental analysis by SEM-EDS of both raw material zeolite and all corresponding synthetized geopolymers are presented in figure 2.

As seen in SEM-EDS images (figure 2), it is clear that there is an increase in C and Na elements attributed to the formation of carbonates and activators used NaOH and sodium silicate, respectively [37].

**Figure 3.**
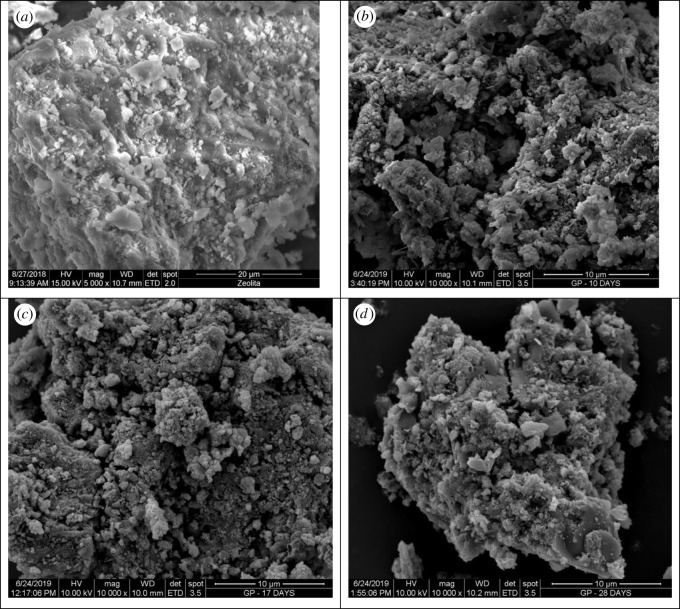
SEM micrographs (*a*) zeolite, (*b*) geopolymer cured for 9 d (G-10), (*c*) geopolymer cured for 16 d (G-17) and (*d*) geopolymer cured for 27 d (G-28).

Figure 3 shows the microstructure of natural zeolite and geopolymer synthesized.

Micrographs demonstrate that especially geopolymer samples have porous structures, which allows them to be used as adsorbents for copper removal from wastewater [3,8]. Bai *et al.* [38] reported that increasing the porosity of the geopolymer adsorbent increases the copper removal capacity and efficiency. The results reported in this current study regarding the porous geopolymer surface and adsorption are consistent with the literature.

### 3.2. Geopolymer compressive strength tests

The compressive strength is one of the most important parameters for geopolymer characterization. It explains how stable is a geopolymer sample under an applied force. Besides, there is a clear relationship between compressive strength and porosity [39]. So, the geopolymers cured for a long time show higher compressive strength and a lesser porosity. Due to this fact, it is important to give compressive strength of a geopolymer sample used for adsorption of copper or any other heavy metal.

Table 2 shows the compressive strength of the geopolymers developed for this study. Additionally, the stress and strain curve of geopolymer samples' compressive strength tests can be seen in electronic supplementary material, figure S5. There is a direct relationship between the curing time and the mechanical properties. Thus, the longer the curing time, the higher the compressive strength obtained. The geopolymer structure's hardening behaviour suggests transforming mordenite and calcite into a load-bearing material as time increases. Besides that, if figure 1 is checked, there is a decreasing tendency in the quantity of quartz and mordenite while increasing amorphous content concerning the curing time. The increase in amorphous content has been proportionally related to the compressive strength and thus better mechanical properties [40]. Cristelo *et al.* [40] reported around 20 MPa of maximum compressive strength for fly ash-based geopolymers cured for 90 d, which is much lower than the maximum compressive strength of 26.86 MPa obtained in the current study. Several studies reported the highest curing days for different compressive strength tests and temperatures: 28 d (15.84 MPa and 50°C) for a mixture of metakaolin, zeolite and cork residues [41]; 14 d (9.95 MPa and 50°C) for a mixture containing metakaolin and zeolite [14]; 28 d (10 MPa at 60°C) for an Ecuadorian zeolite-based geopolymer [10] and 14 d (about 19 MPa at 80°C) for another type of Ecuadorian zeolite-based geopolymer [42]. So, the compressive strength values reported in the current study are higher than some results reported in the literature. The materials that show compressive strength between 13 and 21 MPa are considered construction materials [43]. Due to that, the samples G-17 and G-28, which have presented more than 20 MPa of compressive strength, can easily be considered constriction materials. So, these samples might be used in some potential real-life applications.

**Table 2.** Compressive strengths geopolymer blocks.

sample	curing time (days)	maximum compressive strength (MPa)
G-10	10	17.06
G-17	17	21.35
G-28	28	26.86

### 3.3. Kinetic results

**Figure 4.**
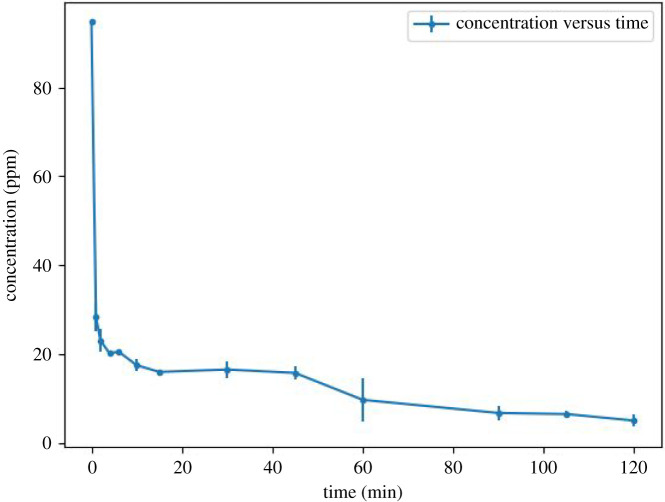
The change in Cu^2+^ concentration in 120 min of contact time with the geopolymer at a batch constant temperature of 25°C.

**Figure 5.**
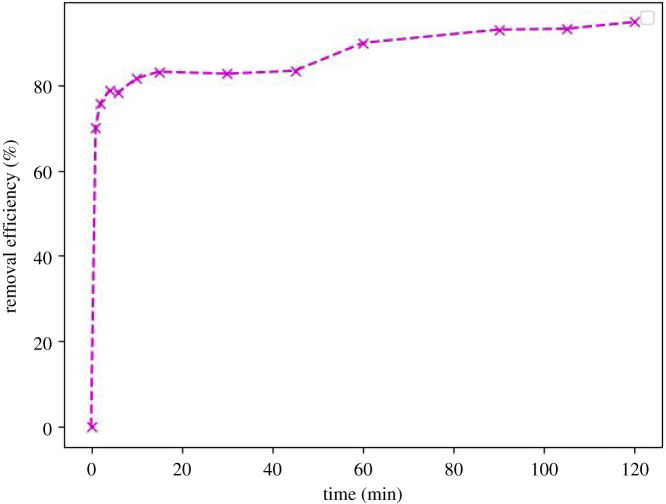
Cu^2+^ removal efficiency of zeolite-based geopolymer for 120 min of contact time at a constant batch temperature of 25°C.

**Figure 6.**
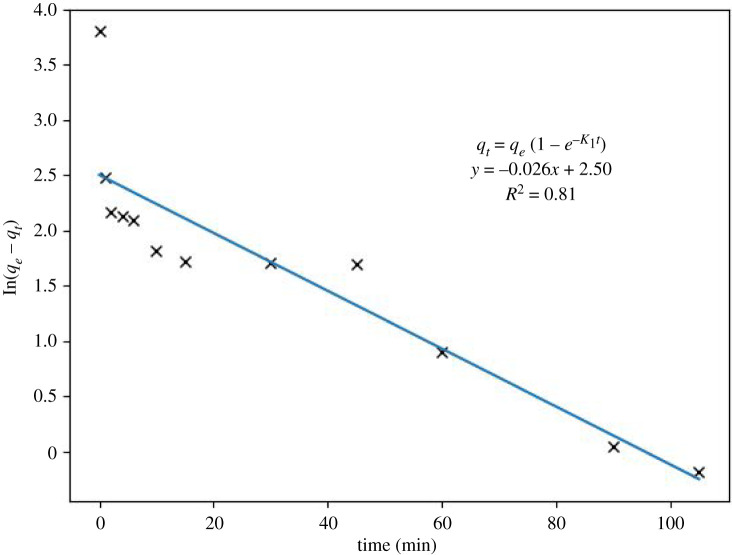
Kinetic results of the pseudo first-order model in Cu^2+^ adsorption for the system that contains initial Cu^2+^ concentration 94.7 ppm for 120 min of contact time and at the constant batch temperature of 25°C.

**Figure 7.**
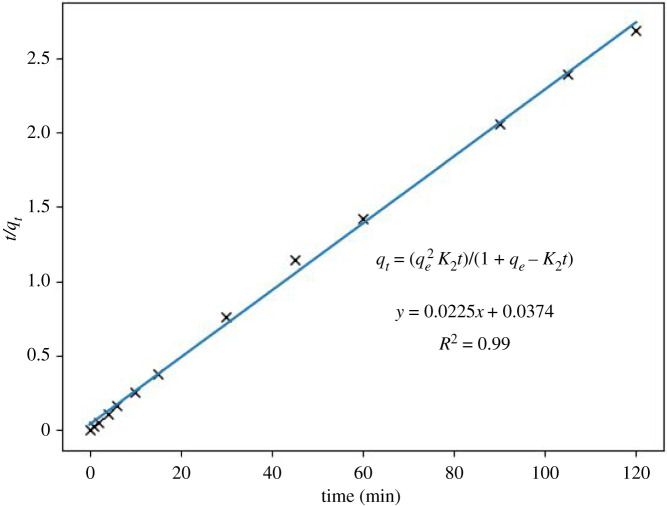
Kinetic results of the pseudo second-order model in Cu^2+^ adsorption for the system that contains initial Cu^2+^ concentration of 94.7 ppm, for 120 min of contact time, and the constant batch temperature of 25°C.

**Figure 8.**
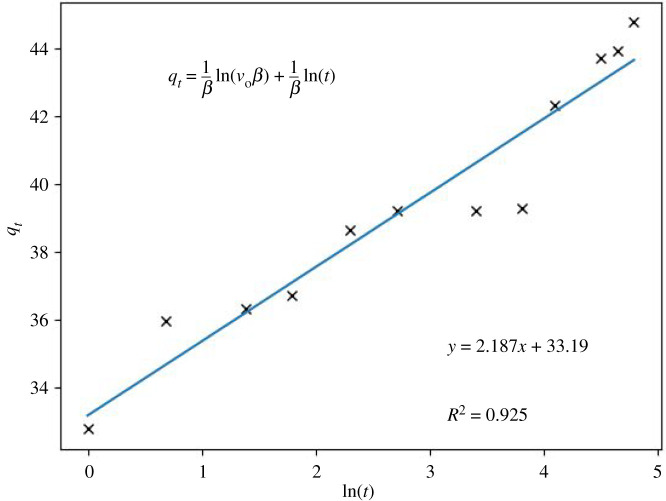
Kinetic results of the Elovich model in Cu^2+^ adsorption for the system that contains initial Cu^2+^ concentration of 94.7 ppm, for 120 min of contact time and, the constant batch temperature of 25°C.

Figures 4 and 5 show the variation of Cu^2+^ concentration plotted versus time and the adsorption efficiency of zeolite-based geopolymer, respectively. It can be seen a step-down in Cu^2+^ concentration within the first minute of contact of the geopolymer with the Cu^2+^ solution, dropping from 94.7 to 28.32 ppm. Then, a slight decrease begins for 2 h, reaching 5.02 ppm of Cu^2+^ in the solution. The kinetic model was determined by the linearization approach using equations (3.1)–(3.3) for pseudo first-order, pseudo second-order and Elovich [44,45], respectively (figures 6–8). The results obtained can be seen in table 3.

3.1 $$q_{t}=q_{e}(1-e^{-k_{1}t}),$$

3.2 $$q_{t}=\frac{q_{e}^{2}k_{2}t}{1+q_{e}k_{2}t}$$

3.3 $$\;\mathrm{and}\;q_{t}=\frac{1}{\beta }\ln(v_{0}\beta )+\frac{1}{\beta }\ln(t),$$

where *q*_e_ (mg g^−1^) is the adsorption capacity at equilibrium, *q*_t_ (mg g^−1^) is the adsorption capacity until *t* (min), *k*_1_ (min^−1^) is the pseudo first-order constant, *k*_2_ (g mg^−1^ min^−1^) is the pseudo second-order constant, *β* (g mg^−1^) is the desorption constant and *v*_0_ (mg g^−1^ *t*^−1^) is the initial adsorption rate.

**Table 3.** Kinetic parameters for adsorption of Cu^2+^ on geopolymer for the initial Cu^2+^ concentration of 94.7 ppm, for 120 min of contact time and at the constant batch temperature of 25°C.

model	parameters	value	unit
pseudo first-order	*R*^2^	0.81	
	residual standard error	16.81 on 10 d.f.	
	*p*-values	6.933 × 10^−5^	
	*K*_1_	0.0262	min^−1^
	*q_e_*	12.21	mg g^−1^
pseudo second-order	*R*^2^	0.99	
	residual standard error	3.566 on 10 d.f.	
	*p*-values	2.217 × 10^−12^	
	*K*_2_	0.0135	g mg^−1^ min^−1^
	*q_e_*	44.44	mg g^−1^
Elovich [44,45]	*R*^2^	0.925	
	residual standard error	0.4649 on 10 d.f.	
	*p*-values	5.99 × 10^−7^	
	*β*	0.4571	g mg^−1^
	*V*_0_	8517998.22	mg g^−1^ min^−1^
Weber–Morris	*R*^2^	0.911	
	residual standard error	1.065 on 10 d.f.	
	*p*-values	5.533 × 10^−7^	
	*K*_id_	0.986	g g^−1^ min^−1/2^
	*C*	34.116	g g^−1^

Given the experimental dataset, linear regression analysis was carried out to fit these data with a mathematical model presenting parameters like *R*^2^. Residual standard error considering the d.f. and *p*-values that best summarize the least square line of relationship between model-based values and time can be seen in table 3. After a careful examination of linear regression parameters, we can say that the pseudo second-order equation is the best for explaining the current dataset. In this case, the least square line for the pseudo second-order equation explains approximately 99% of the variation, its *p*-values are meaningfully lower than 0.05 and residual standard error on 10 d.f. is one of the lowest, i.e. 3.566. When comparing these results with those given in figures 6 and 7, it can be asserted that the adsorption process is better described by the pseudo second-order equation rather than the others.

It must be said that each known theoretical ground of pseudo second-order equation is based on fundamental theories of surface reactions [46]. Therefore, this equation is more accurate as the system reaches equilibrium and diffusion-driven sorption kinetics in non-equilibrium processes [47]. This equation is linked to the direct adsorption/desorption process controlling the overall rate of sorption kinetics [48], which is the present study's case.

Other models, such as the Weber–Morris model [49,50] (equation (3.4)), are based on intraparticle diffusion, which explains that the ion exchange phenomenon is proportional to the square root of time.

3.4 $$q_{t}=K_{\mathrm{id}}t^{0.5}+C,$$

**Figure 9.**
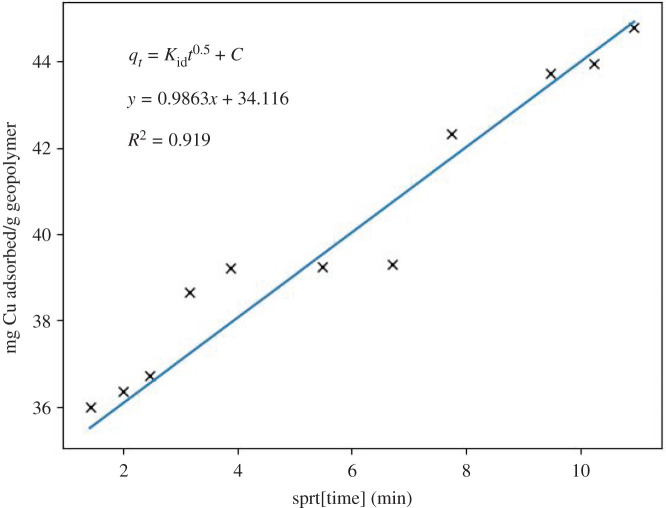
Kinetic results of the Weber–Morris model (intraparticle diffusion model) for the system that contains initial Cu^2+^ concentration of 94.7 ppm, for 120 min contact time and, the constant batch temperature of 25°C.

**Figure 10.**
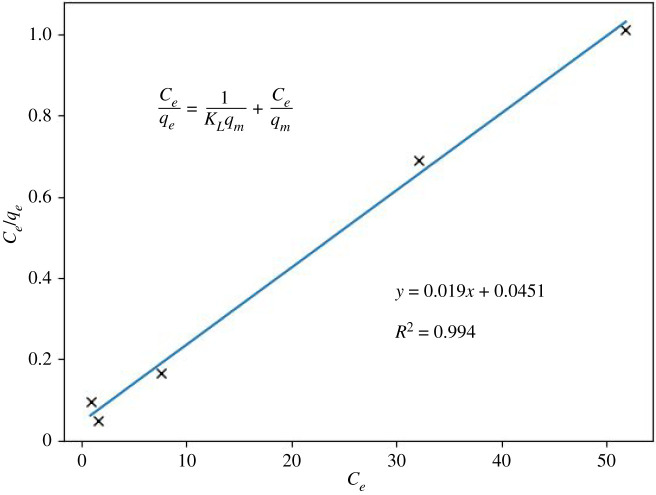
Results of the Langmuir isotherm model (at the constant temperature of 25°C), batch, 2 g l^−1^ dose.

**Figure 11.**
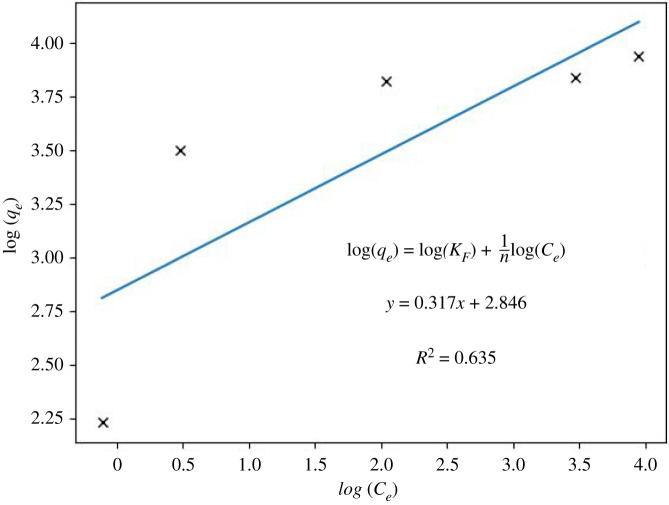
Results of the Freundlich isotherm model (at the constant temperature of 25°C), batch, 2 g l^−1^ dose.

*K*_id_ is the intraparticle diffusion ratio, and *C* is a constant model [51,52]. This model can explain whether the adsorption mechanism occurs on the surface or in the geopolymer's pores. In figure 8, a multilinearity is observed in the diffusion model, indicating a slow ingress of ions into the pores (sodium ion exchange), which appears to be slow, as reflected by the slope. The experimental results brought about for stage *K*_id_: 0,9863 and for C: 34,116 (*R*^2^: 0,9199) (figure 9). Nevertheless, it can be asserted that the Weber–Morris plot for this study indicates that the sorption process consists of several phases. Furthermore, the plots do not pass through the origin, showing that the rate-limiting step is not the pore diffusion (i.e. intraparticle) but the film diffusion (i.e. boundary-layer). Therefore, the first phases of sorption are related to the attachment to the most readily available surface sites, whereas the latter phases involve the slow diffusion of adsorbate from the surface to the inner pores [52]. The correlation value for the Langmuir isotherm model is 0.994 (figure 10), implying a possible formation of monolayers on the adsorbent surface, with a maximum adsorption capacity of 52.63 mg g^−1^. On the other hand, the correlation coefficient value found for the Freundlich isotherm model is 0.635 (figure 11).

**Figure 12.**
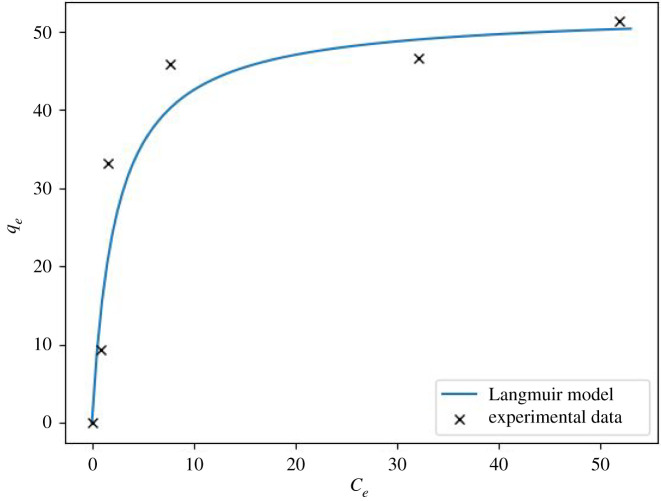
Comparison between experimental data and the Langmuir isotherm for the adsorption at the constant temperature of 25°C, batch, 2 g l^−1^ dose.

Figure 12 represents a comparison of the experimental and theoretical Langmuir isotherm model. This result is consistent with the studies of Singhal *et al*. [33] and Cheng *et al*. [8] which demonstrate that using porous geopolymers for copper removal from water follows the Langmuir model.

Table 4 demonstrates the compared adsorption capacity values for Cu^2+^ adsorption of various studies. Considering adsorption capacity values, the present study shows a competitive adsorption capacity relatively close to that reported by Garcia-Lodeiro *et al.* [30], using metakaolin-based geopolymer spheres.

**Table 4.** Comparison of copper adsorption capacity of the geopolymer under study with other studies.

material	adsorption capacity (mg g^−1^)	reference
metakaolin-based geopolymer	40.9	[8]
metakaolin-based geopolymer	15	[53]
metakaolin-based geopolymer	44.73	[14]
metakaolin-based geopolymer	40	[33]
metakaolin-based geopolymer spheres	35	[54]
metakaolin zeolite-based geopolymer	55.92	[41]
zeolite-based geopolymer	52.63	present study

Elwakeel *et al*. [55,56] reported removal of several metals, including copper, from aqueous media using poly(methyl methacrylate)-Na-Y-zeolite and polyacrylonitrile/Na-Y-zeolite composites. They used adsorption systems that include zeolite and reported very high copper removal efficiency for both composites. Besides, they reported the Langmuir adsorption isotherm and the pseudo second-order kinetics model as the best models, consistently as presented in our study. Even though geopolymers are structurally amorphous, but since they contain zeolite-like structures, aluminosilicates, adsorption and kinetic models found are expected to be similar to the results obtained for zeolite-based systems. So, the consistency of the results between our study and the studies reported by Elwakeel *et al*. [55,56] is the proof for the accuracy of this study.

## 4. Conclusion

This study aims to synthesize Ecuadorian natural zeolite-based geopolymers for a potential industrial application to remove copper from aqueous media. The geopolymers have been prepared using natural zeolite-rich tuffs. The natural Ecuadorian zeolite-rich tuffs were composed of minerals such as quartz (Qz, approximately 21%), mordenite (Mor, 28.5%), calcite (Cc, 4.2%) and 46% of amorphous (Amorph) content. The compressive strength of the geopolymer samples increases with respect to the increasing curing time. The results indicated that the prepared geopolymer samples with a particle size of less than 36 µm is an efficient adsorbent for copper removal compared with similar studies.

Kinetic study reveals that the Cu^2+^ adsorption on the geopolymers follows a pseudo second-order linear behaviour. Consequently, based on the linear coefficient of determination (*R*^2^), the present study is in good agreement with a sorption process of copper on the zeolite-based geopolymer from a very high concentration. Simultaneously, it obeys the pseudo second-order kinetics model at the lower initial concentration of the adsorbate.

Adsorption isotherms calculation results fit perfectly with the Langmuir adsorption model.

## Data Availability

The datasets supporting this article have been uploaded as part of the electronic supplementary material [57].
